# Augmenting Subunit-Vaccine-Induced Immunity through a Dual Strategy of Gold Nanoparticle Conjugation and Chitosan Microneedle-Mediated Sustained Delivery

**DOI:** 10.1021/acsami.5c20082

**Published:** 2025-12-19

**Authors:** Zih-Yao Lin, Yi-Lun Chen, Cheng-Lin Wu, Yu-Hung Chen, Mei-Chin Chen

**Affiliations:** 1 Department of Chemical Engineering, 34912 National Cheng Kung University, Tainan 70101, Taiwan; 2 Department of Pathology, National Cheng Kung University Hospital, College of Medicine, 34912 National Cheng Kung University, Tainan 70403, Taiwan; 3 Institute of Clinical Medicine, College of Medicine, 34912 National Cheng Kung University, Tainan 70101, Taiwan; 4 School of Medicine, College of Medicine, 34912 National Cheng Kung University, Tainan 70101, Taiwan

**Keywords:** adjuvant, antigen depot, chitosan (CS), codelivery, gold nanoparticle (GNP), subunit vaccine, immune activation

## Abstract

Subunit vaccines offer high safety but often exhibit low immunogenicity and rapid clearance and require adjuvants. In this study, we developed a dual strategy for augmenting subunit-vaccine-induced immune responses by integrating self-adjuvanting gold nanoparticle (GNP)-antigen conjugates with implantable chitosan (CS) microneedles (MNs) to achieve sustained intradermal antigen exposure. Conjugation of a model antigen, namely, ovalbumin (OVA), onto the GNP surface (GNP–OVA) resulted in virus-mimicking multivalent antigen display, which substantially enhanced dendritic cell maturation, as evidenced by the upregulation of CD86 and major histocompatibility complex class II. This conjugation strategy also enabled the efficient codelivery of the antigen and carrier into the same antigen-presenting cells, thereby facilitating improved antigen presentation. Furthermore, compared with free OVA and a physical GNP/OVA mixture, conjugated GNP–OVA exhibited considerably longer lymph node retention, primarily because of its nanovaccine properties, which facilitate its preferential trafficking into lymphatic vessels and its subsequent accumulation in lymph nodes. Encapsulation of GNP–OVA into CS MNs (i.e., GNP–OVA MNs) resulted in reliable skin implantation, sustained intradermal antigen exposure, and local immune cell recruitment. Rat immunization studies revealed that GNP–OVA MNs induced balanced T helper 1 and T helper 2 responses and elicited considerably higher and more durable OVA-specific immunoglobulin G levels than did subcutaneous vaccination with GNP–OVA or OVA alone. These responses persisted for at least 16 weeks, highlighting the potential of the developed platform for prolonged subunit vaccine immunization. This dual-strategy platform, combining virus-mimicking GNP-based nanovaccines with immunostimulatory CS MNs, reduces reliance on external adjuvants and enhances the potency and durability of subunit vaccines. Its modular and patient-friendly design underscores its high potential for advancing the development of next-generation vaccines against emerging infectious diseases.

## Graphical abstract

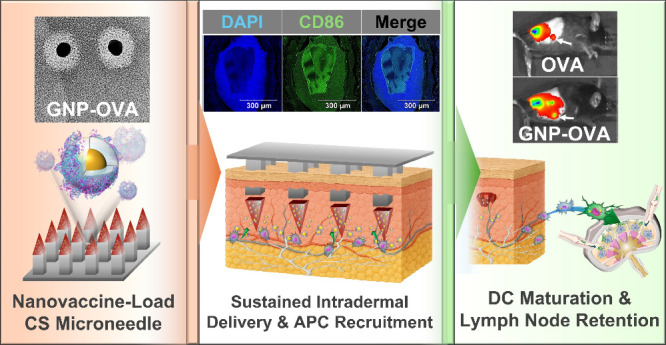

## 1 Introduction

The global burden of infectious diseases, ranging from well-studied pathogens such as hepatitis B virus to rapidly mutating viruses such as influenza viruses and severe acute respiratory syndrome coronavirus 2, continues to pose a persistent and evolving threat to public health.^1–3^ Vaccination is one of the most effective and indispensable strategies for protecting populations and preventing the spread of infectious diseases. Compared with live-attenuated or inactivated vaccines, which are derived from whole pathogens and may carry risks of reversion to virulence, residual pathogenicity, or adverse immune reaction, subunit vaccines offer higher clinical safety.^2–4^ This enhanced safety primarily results from their composition, which involves only antigenic fragments of pathogens, such as proteins, peptides, or polysaccharides, that are specifically selected to induce protective immune responses.^2,3^ Although subunit vaccines exhibit a favorable safety profile, their inherently low immunogenicity necessitates the use of immunostimulatory adjuvants to enhance immune activation.^4,5^ Moreover, their rapid clearance from the injection site and their limited retention in lymphoid tissues may restrict their clinical applicability. These limitations underscore the urgent need to design advanced formulations capable of activating immune cells, prolonging antigen retention, and facilitating lymphatic delivery to improve the efficacy of subunit vaccines.^3,6,7^

Nanoparticles have emerged as promising carriers for peptide- and protein-based vaccines because they can prolong antigen half-life, produce immunostimulatory effects, and ultimately enhance immune responses.^8–10^ Particulate vaccines measuring 10–100 nm tend to exhibit preferential trafficking into lymphatic vessels and subsequent accumulation in lymph nodes. By contrast, smaller particles (<10 nm) or soluble antigens tend to disseminate into the systemic circulation, resulting in poor lymphatic uptake.^8,11,12^ Among various nanomaterials, gold nanoparticles (GNPs) have attracted particular interest because of their unique features, including high biocompatibility, ease of synthesis, tunable size and shape, versatile surface functionalization, and low toxicity.^13–15^ Studies have demonstrated that GNPs can potentiate immune responses by promoting the secretion of cytokines (e.g., interleukin-2, interleukin-6, and tumor necrosis factor-α), enhancing antigen uptake, and stimulating the maturation of antigen-presenting cells (APCs). These effects collectively contribute to the development of both humoral and cellular immunity.^16–19^ On account of their aforementioned attributes, GNPs can serve as both antigen carriers and self-adjuvanting components.

Microneedles (MNs) enable the direct delivery of vaccines into the dermis, which is rich in APCs, offering the potential to elicit stronger immunogenicity compared with conventional intramuscular vaccination.^20,21^ In practice, vaccines are commonly encapsulated within dissolving MNs or coated onto the surface of metallic MNs.^22,23^ Upon the insertion of MNs into the skin, interstitial fluid dissolves the MN tips or surface coating, resulting in the rapid release of the loaded vaccine. However, this bolus administration results in only brief antigen exposure within the skin, which is generally insufficient for inducing durable immunity and robust immunological memory.^24^

This paper proposes two complementary strategies for both overcoming the inherently low immunogenicity of subunit vaccines and inducing long-lasting immune responses: (i) engineering of virus-mimicking vaccines by using self-adjuvanting GNPs and (ii) sustained intradermal delivery of GNP-based vaccines through implantable chitosan (CS) MNs (Figure 1). The model antigen ovalbumin (OVA) is chemically conjugated onto the surface of GNPs (i.e., GNP–OVA) through a thiolated polyethylene glycol (PEG) linker to mimic the size and morphology of viruses. This virus-mimicking design is expected to promote multivalent interactions with APCs and consequently enhance their recognition and uptake by immune cells.^25–27^ Moreover, this GNP conjugation strategy is anticipated to enable the codelivery of abundant antigen and self-adjuvanting GNPs into the same APC, which may facilitate APC maturation and subsequent antigen trafficking to draining lymph nodes for efficient antigen presentation.^28^

**Figure 1:**
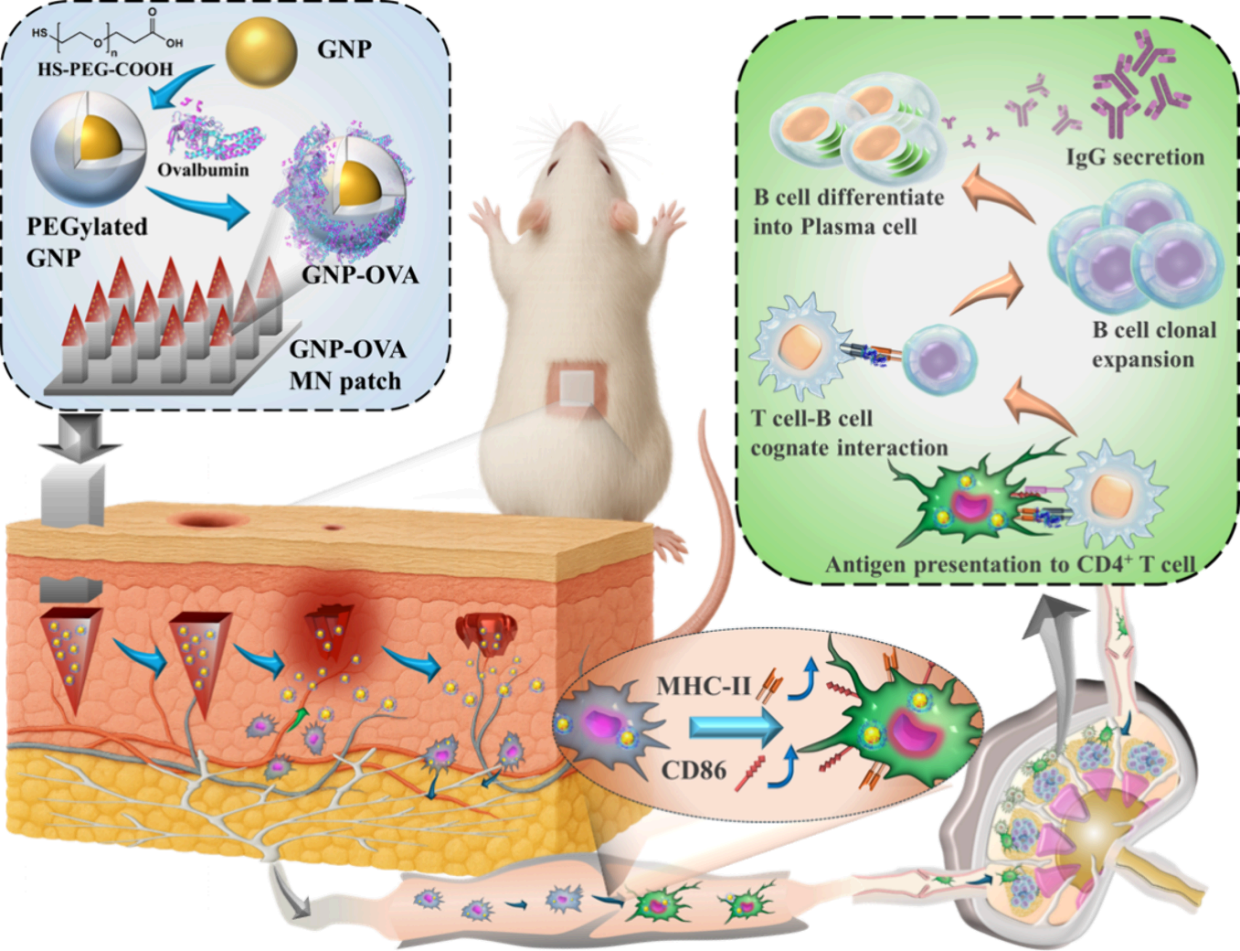
Schematic of a dual-strategy for vaccine delivery. The vaccine delivery system consists of implantable CS MNs loaded with a GNP–OVA nanovaccine and attached to a removable PLA array patch (i.e., GNP–OVA MNs). Upon skin insertion of the CS MNs, the PLA array is detached, leaving the MNs embedded within the dermis to serve as an antigen depot, thereby enabling the sustained release of GNP–OVA through gradual degradation while promoting local immune cell recruitment. Internalized GNP–OVA nanovaccines promote the maturation of DCs and their migration to draining lymph nodes for antigen presentation, leading to enhanced OVA-specific immunoglobulin G production.

To achieve precise and sustained intradermal delivery, the GNP–OVA nanovaccine is encapsulated into biodegradable CS MNs attached to a removable poly­(lactic acid) (PLA) array patch (i.e., GNP–OVA MNs). This extended PLA array ensures the complete insertion of the vaccine-loaded MNs into the dermis and detaches from the skin within 3 min once the adhesion layer between the MNs and the array dissolves in interstitial fluid. Subsequently, the CS MNs remain at the insertion site as an antigen depot for the sustained release of GNP–OVA through gradual degradation.^24,29,30^ Notably, CS has been reported to exhibit adjuvant activity through the recruitment and activation of macrophages and dendritic cells (DCs).^31–33^ Such retention of CS MNs and GNP-based nanovaccines in the skin may enable the prolonged stimulation of immune cells, thereby generating more robust and persistent immune responses.

We previously reported that the conjugation of antigens onto GNPs enhances the uptake and presentation of the antigens by APCs.^26^ Nevertheless, studies have not yet determined whether the extent of antigen conjugation critically influences the immunostimulatory potency of GNP–antigen conjugates and whether the intradermal delivery of these nanovaccines through CS MNs can enhance immune responses by prolonging antigen exposure within the dermis. Therefore, in this study, we examined the effects of the OVA conjugation ratio on DC activation and maturation by assessing the expression of CD86 and major histocompatibility complex class II (MHC-II) molecules. We also compared the cellular codelivery capacity and lymph node retention of the covalently conjugated GNP–OVA nanovaccine to those of a physical GNP/OVA mixture (in which OVA is simply blended with GNPs) and free OVA. The GNP–OVA nanovaccines were encapsulated into CS MNs, after which their intradermal implantation and sustained release capabilities were evaluated in Sprague–Dawley (SD) rats. Finally, an SD rat prime–boost vaccination model was used to compare the OVA-specific immune responses elicited by GNP–OVA MNs with those induced by subcutaneous injections of OVA or GNP–OVA to clarify the individual contributions of GNP conjugation and CS MN delivery to immune augmentation.

## 2 Materials and Methods

### 2.1 Cell Lines, Animals, Materials, and Reagents

The murine dendritic cell line (DC2.4) was obtained from the Bioresource Collection and Research Center (Hsinchu, Taiwan), and female SD rats and C57BL/6 mice, both 8 weeks old, were provided by BioLASCO Taiwan (Taipei, Taiwan) and the National Cheng Kung University (NCKU) Laboratory Animal Center (Tainan, Taiwan), respectively.

Chitosan (>90% deacetylated, 22 mPa·s), 1-ethyl-3-(3-(dimethylamino)­propyl) carbodiimide (EDC, 98%), *N*-hydroxysuccinimide (NHS, 98%), HAuCl_4_·3H_2_O (≥99.9%), poly­(vinyl alcohol) (PVA, MW 10 kDa), polyvinylpyrrolidone (PVP, MW 6 kDa), and ovalbumin (OVA, ≥ 90%) were purchased from Sigma-Aldrich (St. Louis, MO, USA). HS-PEG-COOH (MW 5 kDa) and PLA were obtained from Nanosoft Polymers (Winston-Salem, NC, USA) and Flmt Corp. (Taipei, Taiwan), respectively.

Fluorescein isothiocyanate (FITC)-labeled anti-MHC-II antibody and phycoerythrin (PE)-labeled anti-CD86 antibody were obtained from Elabscience Biotechnology Inc. (Wuhan, China). Anti-CD86 rabbit monoclonal antibody and goat antirabbit IgG H&L (FITC) antibody were obtained from Zen BioScience (Chengdu, China). HRP-conjugated secondary antibodies, including goat antirat IgG2a, goat antirat IgG1, and goat antirat IgG, were purchased from Novus Biologicals (Centennial, CO, USA).

Stainless steel master structures for the MNs and supporting structures were obtained from Hong-Da Precision Industry (New Taipei City, Taiwan).

### 2.2 Ethics Statement

All animal experiments were performed in accordance with the guidelines of the Laboratory Animal Center of NCKU and were approved by the Institutional Animal Care and Use Committee of NCKU (approval no:113156).

### 2.3 Synthesis and Characterization of GNP-OVA

GNP-OVA was synthesized using the citrate reduction method according to our previous work with slight modification.^26^ In brief, 1 mL of 1 wt % tetrachloroauric acid (HAuCl_4_) solution was added to 100 mL of ultrapure water and boiled at 100 °C for 15 min, 6 mL of preheated 1 wt % (34 mM) trisodium citrate dihydrate solution was rapidly added to the mixture and reacted for 20 min to obtain citrate-capped GNP (i.e., naked GNP). After cooling to room temperature (RT), 10 mL HS-PEG-COOH (1 mg/mL) solution was added to the as-prepared GNP solution to form GNP-PEG via Au–S bond. The mixture was then incubated in an orbital shaker at 250 rpm at RT overnight to complete ligand exchange. One milliliter of GNP-PEG solution was purified by centrifugation (20000 g, 30 min) twice and then resuspended in 0.8 mL MES buffer (pH 5.6), and different concentrations (Table S1) of 0.1 mL EDC and 0.1 mL NHS were added to each group according to different OVA/GNP (O/G) conjugation ratios. After activating for 15 min, the mixture was centrifuged and resuspended in 0.8 mL PBS (pH 7.2). OVA solutions at different concentrations (Table S1) were then added to each activated GNP-PEG solution and allowed to react for 4 h. The resulting GNP-OVA solutions were washed three times with PBS by centrifugation (20,000 g) and redispersion. The synthesized GNP-OVA solution was stored at 4 °C before further used.

The grafted OVA amount was determined by subtracting the unreacted OVA, quantified by bicinchoninic acid (BCA) assay, from the initial OVA feed. The morphology of the GNP-OVA conjugates was characterized using transmission electron microscopy (TEM; H7500, Hitachi, Tokyo, Japan). TEM samples were prepared by casting 10 μL particle solution onto carbon-coated copper grids stained with 1 wt % phosphotungstic acid solution. The GNP concentrations in the test samples were quantified using inductively coupled plasma mass spectrometry (ICP-MS; Element XR, Thermo Fisher Scientific, San Jose, CA, USA). The hydrodynamic diameter and polydispersity index (PDI) were determined by dynamic light scattering (DLS; Zetasizer Nano ZS90, Malvern Instruments, Malvern, UK).

### 2.4 Cell Viability Assay

The DC2.4 cells were cultured in RPMI 1640 medium containing 10% (v/v) FBS and 1% (v/v) penicillin–streptomycin. Cells were seeded in 96-well plates at a density of 4 × 10^4^ cells/well and incubated for 2 days. After washing with PBS, the cells were treated with test samples, positive control (PBS), or negative control (70% ethanol) for 12 h. The GNP-OVA solutions were sterilized by filtration through a 0.22-μm syringe filter and mixed with fresh medium at a ratio of 1:9, ensuring that all groups contained the same GNP concentration (400 μg/mL). Cell viability was quantitatively evaluated using the PrestoBlue assay.

### 2.5 Flow Cytometric Analysis of DC Activation and Maturation: CD86 and MHC-II

DC2.4 cells were seeded in 12-well plates at a density of 2 × 10^5^ cells/well and incubated for 2 days. The OVA concentration in all test groups, except the PBS control, was adjusted to 25 μg/mL by mixing with fresh medium at a ratio of 1:9. After 12 h coculture, the cells were harvested and stained with FITC-labeled anti-MHC-II and PE-labeled anti-CD86 antibody according to the manufacturer’s instructions. The stained cells were washed with PBS, fixed with paraformaldehyde, and analyzed by flow cytometry using a CytoFLEX flow cytometer (Beckman Coulter, Brea, CA, USA).

### 2.6 Co-delivery of GNP and Antigens to DC2.4

DC2.4 cells were seeded in 35 mm dishes at a density of 1.6 × 10^5^ cells/dish and cultured for 2 days. Cells were then treated with OVA, a physical mixture of GNP and OVA (GNP/OVA) or GNP-OVA conjugates at fixed concentrations of GNP (200 μg/mL) and OVA (25 μg/mL) for 4 h. After treatment, the cells were washed with PBS, fixed with paraformaldehyde, and mounted with 4′,6-diamidino-2-phenylindole (DAPI) antifade medium, after which intracellular distribution of GNP and OVA was examined using an FV3000 confocal laser scanning microscope (Olympus, Tokyo, Japan). For fluorescence visualization, GNPs were labeled with Cy3 (Cy3-GNP) and OVA with Cy5 (Cy5-OVA). The detailed procedures for preparing Cy3-GNP, Cy5-OVA, and Cy3-GNP-OVA-Cy5 are provided in the Supporting Information **.**

### 2.7 *In Vivo* Lymph Node Drainage Tracking

To investigate the in vivo lymphatic drainage and retention of GNP-OVA, female C57BL/6 mice were subcutaneously injected at the dorsal site near the base of the tail with 100 μL of the test formulations containing a fixed OVA concentration (1 mg/mL). For fluorescence visualization, OVA was labeled with Cy7 (Cy7-OVA). Images were acquired at predetermined time points using an in vivo imaging system (IVIS; PerkinElmer, Waltham, MA, USA). Regions of interest (ROIs) of identical size were drawn over the draining inguinal lymph nodes in all groups to ensure quantitative comparability, and the total radiant efficiency ([photons/sec/cm^2^/sr]/[μW/cm^2^]) was quantified using Living Image software (PerkinElmer, Waltham, MA, USA).

### 2.8 Fabrication of GNP-OVA MN Patches

The nanovaccine delivery patch was fabricated by integrating GNP-OVA-loaded CS MNs with a PLA array patch (Figure S1). First, 3.2 g of CS powder was dissolved in 100 mL of 1% (v/v) acetic acid and dialyzed against deionized water for 3 days until pH reached ∼ 6.0. Trehalose (16 mg) was then added to the resulting 1.6 wt % CS solution (10 mL), which was further concentrated at 75 °C to obtain a 5 wt % CS solution. Subsequently, 1.5 mL of GNP-OVA solution (7 mg/mL) was added to the CS solution, thoroughly mixed, and further concentrated at room temperature under stirring to obtain a 10 wt % CS gel containing GNP-OVA.

Approximately 30 mg of the gel was cast onto a polydimethylsiloxane (PDMS) MN mold and covered with a polytetrafluoroethylene (PTFE) plate. A compression force of 300 N was applied to the PTFE plate for 30 s using a universal testing machine (AGS-500NX, Shimadzu, Kyoto, Japan) to ensure complete filling of the mold cavities. Excess gel was then removed from the mold surface, followed by drying in an oven at 37 °C for 8 min. A PLA pressing tool was subsequently employed to compact the semidried gel into the mold tips. This filling-pressing cycle was repeated five times, after which the molds were dried at 37 °C for 1 h to yield GNP-OVA-loaded CS MNs within the molds.

To integrate the MNs with the PLA array patch, a 50 wt % aqueous solution of PVP and PVA (1:1, w/w) was used as an adhesive. An aliquot of the PVP/PVA solution (0.3 mL) was applied onto the surface of the MNs-containing mold, which was subsequently placed in a vacuum oven at 0.2 atm for 6 min. After removal of excess adhesive, a prefabricated PLA array patch was carefully aligned and pressed into the mold cavities to attach to the CS MNs. The patches were dried overnight at room temperature and gently demolded.

### 2.9 Quantification of GNP-OVA Amount per MN Patch

The GNP-OVA content in the MN patches was determined by quantifying the GNP amount using ICP–MS, followed by calculation of the total GNP-OVA content based on the predetermined OVA/GNP conjugation ratio. For GNP quantification, the GNP-OVA MN patches were immersed in 0.75 mL of 37% HCl and stirred at 70 °C to achieve complete dissolution of CS. After cooling to room temperature, 0.25 mL of 67% HNO_3_ was added, and the mixture was stirred for 2 h. The resulting solution was then diluted 10-fold with ultrapure water prior to ICP-MS analysis.

### 2.10 Mechanical Strength and Skin Insertion of MNs

A universal testing machine was employed to measure the failure force and force–displacement behavior of the MNs. Each MN patch was mounted on the machine with the needles facing upward and compressed at a rate of 1 mm/min (n = 4 patches).

To evaluate the skin insertion capability, MN patches were inserted into porcine abdominal skin in vitro or rat dorsal skin in vivo using a spring-loaded applicator (10 N/patch, 3 min). For in vivo studies, rat dorsal hair was removed before application. Skin samples were then excised, embedded in OCT, and cryosectioned with a cryostat (CM1860, Leica Biosystems, Nussloch, Germany) to assess insertion depth. The insertion depth was quantitatively analyzed by measuring all MNs embedded within five histological cross sections.

### 2.11 In Vitro Release Analysis Using a Franz Diffusion Cell System

The in vitro release of GNP-OVA from MN patches was assessed using a Franz diffusion cell system. Briefly, MN patches were applied to porcine cadaver skin and mounted between the donor and receptor compartments. The receptor chamber was filled with 4 mL of PBS (pH 7.4) and maintained at 37 °C under continuous stirring. At predetermined intervals, the entire receptor medium was withdrawn and replaced with an equal volume of fresh PBS. The cumulative release of GNP was quantified by ICP-MS after centrifugation (20,000 × g, 1 h).

### 2.12 In Vivo Retention of GNP-OVA in Rat Skin

The retention of GNP-OVA released from the MN patches was investigated in SD rats. GNP-OVA MN patches (160 μg of GNP-OVA-Cy7) were applied onto the dorsal skin, whereas an equivalent dose of GNP-OVA solution was administered by subcutaneous injection. Fluorescence signals were acquired at predetermined time points using an IVIS. ROIs with identical size were drawn over the administration sites across all groups to ensure quantitative comparability. Data were analyzed with Living Image software as described in Section 2.7.

### 2.13 Histological and Immunofluorescent Analysis of Immune Cell Recruitment

Hematoxylin and eosin (H&E) staining and immunofluorescence (IF) analysis were performed to evaluate whether MN application induced local immune cell recruitment. SD rats were randomly divided into three groups: PLA MN (Sham), CS MN, and GNP-OVA MN. Following MN insertion, skin tissues from the administration sites were excised, sectioned, and subjected to H&E staining. For IF staining, sections were deparaffinized and rehydrated, followed by heat-induced antigen retrieval in citrate buffer (pH 6.0). The sections were then incubated with an anti-CD86 primary antibody, followed by an FITC-conjugated IgG secondary antibody. Samples were mounted with DAPI-containing antifade medium. Fluorescence images were acquired using an FV3000 confocal laser scanning microscope.

### 2.14 Rat Immunization and Specific Immunoglobulin G Levels Analysis

SD rats were randomly divided into four groups: (i) PBS group, (ii) OVA group (80 μg/rat), and (iii) GNP-OVA group (160 μg/rat, comprising 80 μg OVA and 80 μg GNP), all of which received subcutaneous injections; and (iv) GNP-OVA MN group, treated with MN patches containing the same dose of GNP-OVA conjugates. All groups were immunized at the dorsal skin on day 0 and boosted on day 14. Blood samples were collected from the submandibular vein after immunization. Serum was isolated by centrifugation (3000 × g, 10 min, 4 °C) and stored at – 20 °C until analysis. OVA-specific IgG, including total IgG, IgG1, and IgG2a, was quantified by enzyme-linked immunosorbent assay (ELISA) at serum dilutions of 1:8000, 1:1000, and 1:1000, respectively, as described previously.^26^

### 2.15 Statistical Analysis

Statistical analyses were performed using one-way analysis of variance (ANOVA). Data are presented as mean ± standard deviation (SD). A p-value <0.05 was considered statistically significant.

## 3 Results and Discussion

### 3.1 Characterization of GNP–OVA Conjugates

To enhance the immunogenicity of subunit vaccines, we developed specific GNP–antigen conjugates designed to mimic the size and multivalent surface presentation characteristic of natural viruses. Although these are not classical virus-like particles derived from viral proteins, we hypothesized that these virus-mimicking nanoparticles could directly interact with APCs and facilitate efficient antigen uptake and presentation. To prepare the GNP–antigen nanovaccines, GNPs were synthesized through citrate reduction. Their surface was then functionalized with HS–PEG–COOH through Au–S bonding, which displaced surface-bound citrate anions, permitting OVA conjugation (Figure 2a) and preventing GNP aggregation.^34^ Next, the resulting GNP–PEG was conjugated with OVA via the EDC/NHS coupling reaction, in which the activated carboxyl groups of GNP–PEG reacted with the primary amine groups of OVA.

**Figure 2:**
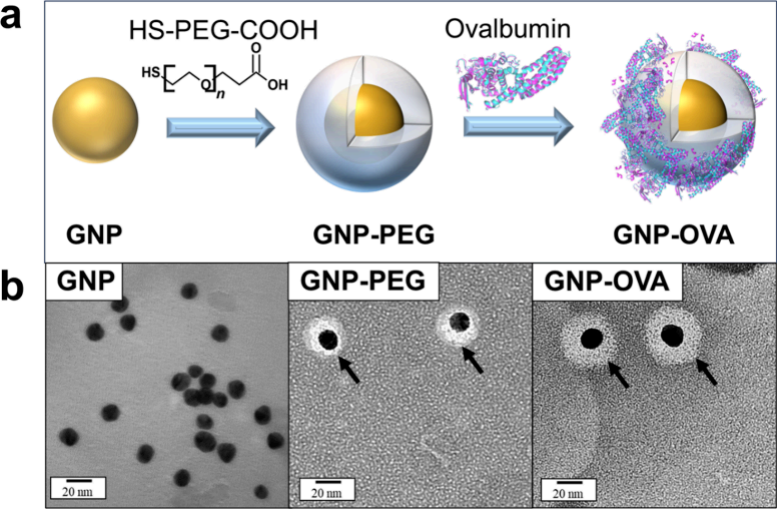
Schematics and TEM images of GNP, GNP–PEG, and GNP–OVA. (a) Preparation of GNP–OVA nanoconjugates through surface modification of GNPs with carboxyl-terminated PEG (HS–PEG–COOH), followed by the covalent conjugation of OVA. (b) TEM images of GNP, GNP–PEG, and GNP–OVA. The arrows in the TEM images of GNP–PEG and GNP–OVA point toward the shell and corona-like structures around GNPs, respectively. Scale bars: 20 nm.

TEM images revealed that the synthesized GNPs exhibited a nearly spherical morphology with a narrow size distribution (Figure 2b). The average hydrodynamic diameter and polydispersity index (PDI) of these GNPs were 15.4 ± 4.5 nm (*n* = 5) and 0.17 ± 0.01 (*n* = 5), respectively (Table 1). As expected for citrate-stabilized nanoparticles, the GNPs exhibited a highly negative zeta potential (−39.0 ± 1.9 mV, *n* = 4), which was attributable to the adsorption of citrate anions onto the nanoparticle surface. After PEGylation, GNP–PEG displayed a distinct core–shell morphology (Figure 2b), with the gold core surrounded by a uniform, lighter PEG shell. This observation was consistent with DLS measurements, which indicated that the particle size markedly increased to 26.0 ± 5.4 nm (*n* = 5, Table 1). Moreover, the zeta potential became less negative (−33.9 ± 1.2 mV, *n* = 4), reflecting the replacement of citrate by thiolated PEG linkers with higher affinity for the gold surface.

**Table 1:** Quantity of Conjugated OVA (*n* = 8), Size Distribution, PDI (*n* = 5), and Zeta Potential (*n* = 4) for GNP–OVA Conjugates^a^

	OVA_conj_ (μg/mL)	GNP (μg/mL)	size (nm)	PDI	zeta potential (mV)
**O/G = 1**	84.7 ± 5.7	88	41.1 ± 8.9	0.30 ± 0.03	–21.1 ± 1.8
O/G = 1/2	44.7 ± 3.4	88	35.7 ± 7.8	0.29 ± 0.03	–24.8 ± 2.1
**O/G = 1/4**	21.9 ± 0.8	88	31.2 ± 7.1	0.27 ± 0.02	–25.7 ± 2.2
**O/G = 1/8**	10.6 ± 0.9	88	28.7 ± 6.0	0.22 ± 0.02	–29.2 ± 1.7
**GNP-PEG**	N/A	N/A	26.0 ± 5.4	0.19 ± 0.01	–33.9 ± 1.2
**GNP**	N/A	N/A	15.4 ± 4.5	0.17 ± 0.01	–39.0 ± 1.9

a The quantity of conjugated OVA (OVA_conj_) was calculated by subtracting the unreacted OVA in the supernatant, determined through a BCA assay, from the initial OVA feed. O/G: OVA/GNP conjugation ratio.

Upon OVA conjugation, the uniform core–shell morphology of GNP–PEG transformed into a corona-like structure (Figure 2b), accompanied by an increase in the hydrodynamic diameter to 41.1 ± 8.9 nm (*n* = 5). The surface charge shifted toward less negative values as the degree of OVA conjugation increased, ultimately reaching – 21.1 ± 1.8 mV (*n* = 4) for an O/G conjugation ratio of 1. This value approaches the zeta potential of free OVA (−13.1 ± 1.1 mV), indicating the successful conjugation of OVA onto the GNP surface. Overall, the observed morphological evolution, size increments, and zeta potential shifts strongly support stepwise surface modification from citrate-capped GNPs to PEGylated GNPs and then OVA-conjugated GNPs.

Nanoparticles conjugated with antigens on the surface can mimic multivalent antigen display, enabling the cross-linking of immune cell surface receptors.^35,36^ The quantity of antigens conjugated onto the nanoparticle surface has a critical effect on their interaction with APCs, which in turn influences APC activation and subsequent immune responses. In this study, GNP-based nanovaccines with varying OVA conjugation ratios were prepared by adjusting the reaction concentrations of OVA and EDC/NHS (Table S1), and their effects on DC maturation were then evaluated. As presented in Table S2, the quantity of conjugated OVA (OVA_conj_) increased in proportion with the OVA feed concentration (OVA_in_), confirming the tunability of the quantity of conjugated antigens. Moreover, the particle size of GNP–OVA conjugates slightly increased as the O/G conjugation ratio increased (Table 1 and Figure S2).

To evaluate the cytocompatibility of the GNP–OVA nanovaccines, DC2.4 cells were cocultured with GNP–OVA of varying O/G ratios at a fixed GNP dose (400 μg/mL) for 12 h. As displayed in Figure S3, all GNP–OVA groups maintained cell viability above 90%, demonstrating no significant difference compared with the PBS control (*p* > 0.05, *n* = 6). These results confirmed that the GNP–OVA nanovaccines were cytocompatible, supporting their use in subsequent DC activation assays and rat immunization studies.

### 3.2 Effects of the O/G Ratio on CD86 and MHC-II Expression in DCs

DCs are the most potent APCs, playing a central role in the initiation of adaptive immune responses essential for host defense and vaccine-induced immunity.^35,37^ To further investigate how the O/G ratio influences APC activation, we evaluated CD86 and MHC-II expression in DC2.4 cells treated with GNP-based nanovaccines having varying O/G ratios for 12 h. CD86, a costimulatory molecule typically upregulated on mature DCs, provides the essential “second signal” for T-cell priming, whereas MHC-II molecules function as an antigen-presenting platform for the recognition of CD4^+^ T cells. These markers were selected as indicators of DC maturation and antigen-presenting capacity (Figure 3).

**Figure 3:**
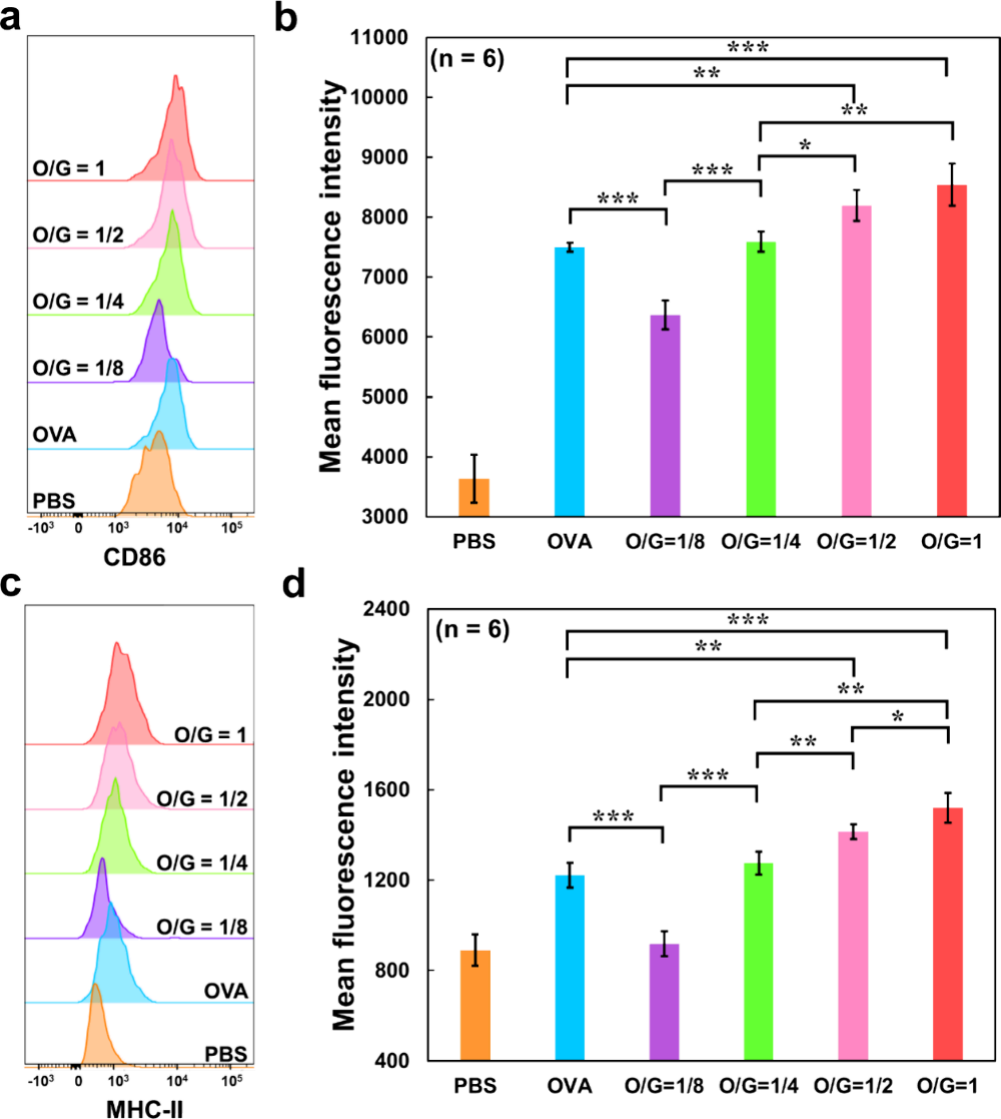
Flow cytometric analysis results of CD86 and MHC-II expression in DC2.4 cells treated with GNP–OVA nanovaccines at different O/G ratios. (a, c) Representative histograms and (b, d) mean fluorescence intensities corresponding to (a, b) CD86 and (c, d) MHC-II expression after 12 h of incubation with free OVA, GNP–OVA nanovaccines at different O/G ratios (1/8, 1/4, 1/2, and 1), or PBS (used as a negative control). The OVA dose was fixed at 25 μg/mL across all groups. Data are presented as mean ± standard deviation (*n* = 6). *p < 0.05; **p < 0.01; ***p < 0.001.

Flow cytometric analysis revealed marked upregulation of CD86 and MHC-II expression as the O/G ratio increased from 1/8 to 1, indicating that GNPs conjugated with a higher quantity of antigens promote DC maturation and antigen presentation more effectively. Notably, GNP–OVA nanovaccines with an O/G ratio of ≥ 1/2 induced significantly higher CD86 and MHC-II expression compared with free OVA (*p* < 0.05, *n* = 6). This result suggests that, compared with the monovalent binding of a single antigen, antigen display on nanoparticles at an optimal ratio can promote multivalent interactions with APCs, thereby enhancing APC activation and subsequent molecular events and ultimately eliciting stronger immune responses.^38^ Moreover, the specimen group with O/G = 1/8 exhibited lower CD86 and MHC-II expression compared with the free OVA group (*p* < 0.05, *n* = 6). This result was attributable to the OVA dose being fixed across all groups; thus, lower O/G ratios corresponded to higher quantities of GNPs in the medium, which likely resulted in excessive nanoparticle uptake, leading to phagocytic overload and lysosomal stress and ultimately impairing APC function.^39^ Accordingly, the GNP–OVA group with an O/G ratio of 1, which exhibited the strongest capacity to promote DC activation and antigen presentation among all specimen groups, was selected for subsequent investigations. Figure S4 depicts the percentages of CD86^+^ and MHC-II^+^ cells for each group.

### 3.3 Co-delivery of Antigen and Self-Adjuvanting GNPs to DCs

Co-delivery of antigens and adjuvants to the same APCs is essential for effective immune activation because it enables the simultaneous recognition of the antigens and adjuvants by immune cells, which in turn elicits a rapid, antigen-specific immune response.^40^ To evaluate whether GNP–OVA conjugates can efficiently mediate such codelivery, DC2.4 cells were incubated with Cy5–OVA, a physical mixture of Cy3–GNP and Cy5–OVA (GNP/OVA), or Cy3–GNP–OVA–Cy5 conjugates (GNP–OVA). As displayed in Figure 4, DC2.4 cells treated with Cy5–OVA alone displayed minimal intracellular fluorescence, indicating limited antigen uptake. For the physical mixture, Cy5–OVA and Cy3–GNP signals were observed from spatially separated vesicular compartments, reflecting unequal and independent uptake of OVA and GNPs. By contrast, cells exposed to the GNP–OVA conjugates exhibited considerably high intracellular accumulation of both Cy5–OVA and Cy3–GNP, with extensive signal overlap (white arrows in the merged image in Figure 4). Taken together, these findings indicate that the proposed conjugate design enables the efficient codelivery of antigens and adjuvants into the same cells, as evidenced by a semiquantitative analysis of the spatial correlation between OVA and GNPs in DCs (Figure S5).

**Figure 4:**
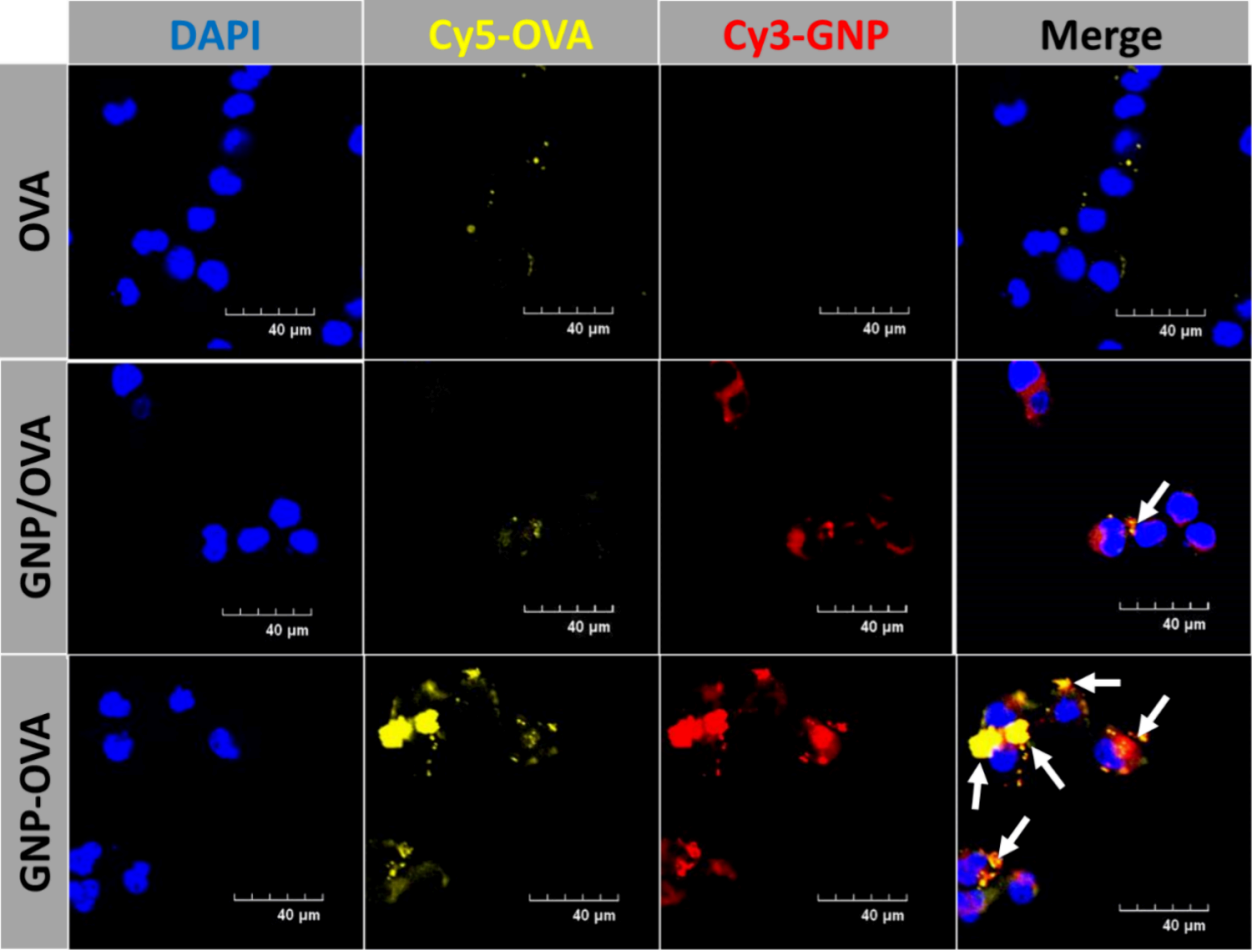
Confocal microscopy images of the intracellular uptake and distribution of OVA and GNPs in DC2.4 cells. Cells were incubated for 4 h with Cy5–OVA (yellow), a physical mixture of Cy3–GNPs (red) and Cy5–OVA (GNP/OVA), or Cy3–GNP–OVA–Cy5 conjugates (GNP–OVA). Nuclei were stained with DAPI (blue). The white arrows indicate intracellular regions demonstrating overlapping Cy5–OVA and Cy3–GNP fluorescence signals. Scale bars: 40 μm.

In this study, the conjugated formulation not only enhanced the intracellular accumulation of OVA but also likely protected it against premature lysosomal degradation, thereby enabling a greater proportion of intact antigen to be processed for MHC-II molecule presentation. Overall, these findings indicate that chemical conjugation offers distinct advantages over physical mixing by preserving antigen integrity and synchronizing immune signals, both of which are pivotal for efficient DC activation and subsequent T-cell priming.^18,41^

### 3.4 Lymph Node Drainage and Retention

Lymph node targeting is a key factor for assessing vaccine carrier performance because lymph nodes serve as central hubs for antigen presentation and the initiation of adaptive immune responses. Antigens reach lymph nodes through two main pathways: (i) passive transport and (ii) active cell-mediated transport. Passive transport involves antigens diffusing with interstitial fluid into the initial lymphatics, whereas active cell-mediated transport involves APCs, predominantly DCs, capturing antigens at the injection site and subsequently migrating to draining lymph nodes in response to chemokine signals.^42^ In this study, to evaluate lymph node drainage and retention, C57BL/6 mice were subcutaneously injected with Cy7–OVA (OVA), a physical GNP/Cy7–OVA (GNP/OVA) mixture, or GNP–OVA–Cy7 (GNP–OVA) conjugates, and fluorescent signals at inguinal lymph nodes were monitored using IVIS.

As shown in Figure 5, all specimen groups exhibited comparable total radiant efficiency at 3 h after injection, reflecting the initial passive diffusion of antigen. Notably, from 6 to 48 h after injection, the GNP–OVA group consistently demonstrated greater accumulation in lymph nodes compared with the GNP/OVA mixture and free OVA groups. This prolonged retention was attributable to the GNP conjugation strategy, which protected OVA against rapid degradation and facilitated its uptake by the same APCs, thereby extending antigen availability for passive lymphatic entry and promoting cell-mediated antigen trafficking to draining lymph nodes. In addition, the GNP/OVA mixture group exhibited significantly stronger signals compared with the free OVA group (*p* < 0.05) after 24 h, suggesting that even physical blending with GNPs inherently aids antigen transport to lymph nodes, likely through enhanced immune cell recruitment and cell-mediated trafficking.^43^ Together, these findings indicate that the chemical conjugation of antigen to GNPs is an effective strategy for enhancing the lymph node delivery and retention of antigen.

**Figure 5:**
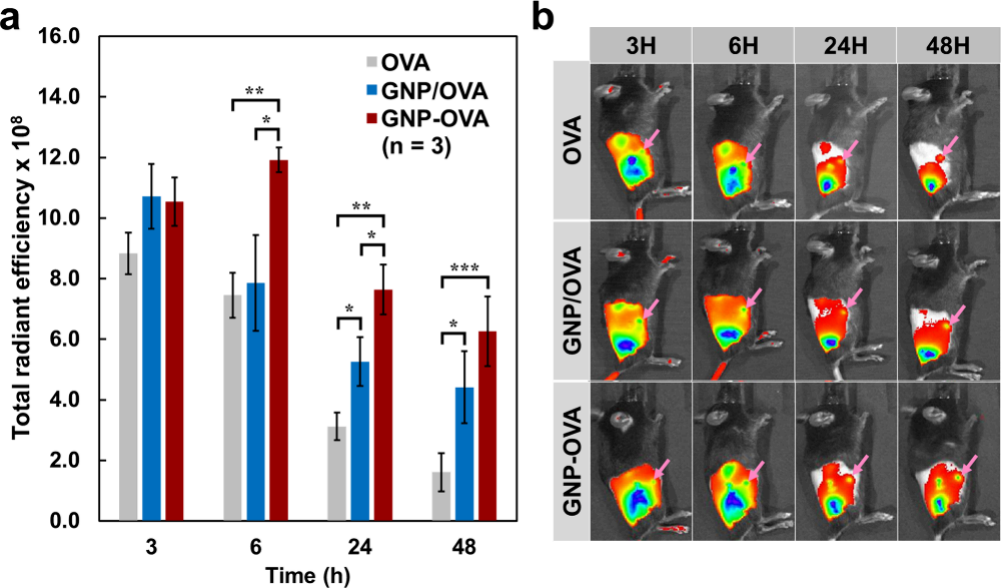
In vivo lymphatic drainage and retention of GNP–OVA in C57BL/6 mice. Cy7–OVA, a physical mixture of GNP and Cy7–OVA (GNP/OVA), or GNP–OVA–Cy7 conjugates (GNP–OVA) were subcutaneously injected into the lower back of each mouse (100 μg of OVA per mouse). (a) Quantitative analysis results of total radiant efficiency at the inguinal lymph node over 48 h. (b) Representative IVIS-generated fluorescence images at 3, 6, 24, and 48 h after injection (the pink arrows indicate the inguinal lymph node site). Data are presented as mean ± standard deviation (*n* = 3). *p < 0.05; **p < 0.01; ***p < 0.001.

### 3.5 Characterization and Skin Insertion of GNP–OVA MNs

To enable the sustained intradermal delivery of nanovaccines, GNP–OVA conjugates were encapsulated into CS MNs and integrated with a PLA array patch (GNP–OVA MNs). CS was used as the MN matrix because of its inherent biocompatibility, biodegradability, and immunostimulatory properties.^9,14,44^ This PLA array was designed to provide the mechanical strength required for the reliable and complete insertion of the MNs into the dermis. Its detachable configuration enabled it to be rapidly removed from the skin within 3 min, leaving the CS MNs embedded in situ. This removable design not only minimizes patient discomfort and skin irritation but also improves user compliance, making the system more suitable for practical vaccination applications.

Notably, the fabricated array patch (10 mm × 10 mm) contained 9 × 9 MNs arranged with a center-to-center spacing of 1000 μm between adjacent needles (Figure 6 **a, b**). Each pyramidal CS MN and the cuboidal PLA array had a base width of 300 μm and a height of 600 μm. The GNP–OVA nanovaccine (dark red) was successfully encapsulated and uniformly distributed within the MN region, which ensured consistent dosing across all needles.

**Figure 6:**
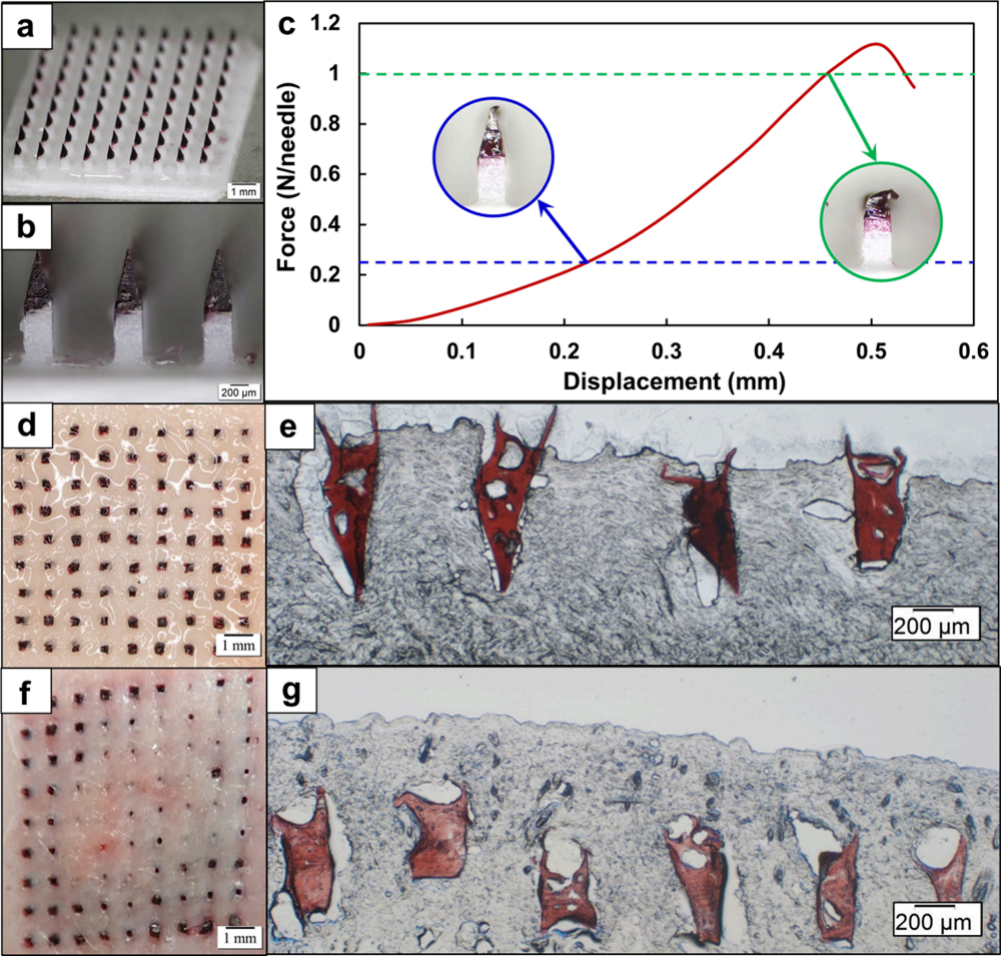
Characterization and skin insertion of GNP–OVA MNs. (a, b) Optical micrographs of the fabricated array patch. (c) Force–displacement curve of the GNP–OVA MN patch. The left inset depicts slight tip bending under a compressive force of 0.25 N per needle, whereas the right inset displays pronounced deformation under a compressive force of 1.0 N per needle. (d) Microwounds on the surface of porcine cadaver skin after MN application and (e) a corresponding cryosection micrograph. (f) Microwounds on the surface of rat dorsal skin after MN application and (g) a corresponding cryosection micrograph. Scale bars: 1 mm in panels (a), (d), and (f) and 200 μm in panels (b), (e), and (g).

A universal testing machine was used to evaluate the mechanical strength of the GNP–OVA MNs by applying a compressive force on the MN array and measuring their resistance to deformation or failure. As displayed in Figure 6c, under a compressive force of 0.25 N per needle, only minor tip bending was observed without structural fracture. More pronounced deformation occurred under a compressive force of 1.0 N per needle. However, the GNP–OVA MNs exhibited a maximum failure force of approximately 1.1 N per needle, which is substantially higher than the minimum force required for skin puncture (0.1 N per needle).^45^ These results indicated that the MNs had sufficient mechanical robustness to ensure reliable skin penetration without being fractured.

After the MN patch was applied to porcine cadaver skin and rat dorsal skin, visible microwounds were observed on the skin surface (Figure 6 **d, f**). The insertion ratio, calculated by dividing the number of microwounds by the total number of needles per patch, reached 100% (*n* = 6) in porcine skin and 97.7% ± 2.2% (*n* = 6) in rat skin (Figure 6 **d, f**). These high insertion ratios demonstrated the reliable mechanical strength of the PLA-supported CS MNs, which could ensure consistent skin penetration across different types of tissue. Such reliable insertion is essential for reproducible intradermal delivery and effective immunization.

The GNP–OVA MNs were successfully implanted into the skin, with average insertion depths of 593.6 ± 97.0 μm (*n* = 5) and 897.9 ± 96.6 μm (*n* = 5) achieved in porcine skin and rat skin, respectively (Figure 6 **e, g**). These depths are well within the dermal layer, which is densely populated with APCs, thereby providing favorable conditions for efficient antigen capture and DC activation. Such localization may facilitate robust adaptive immune responses.

### 3.6 In Vitro GNP–OVA Release from CS MNs

To evaluate the antigen release profiles, GNP–OVA MNs were applied to porcine cadaver skin, and nanovaccine permeation across the skin was examined using a Franz diffusion cell system (Figure 7a). The average GNP–OVA loading per MN patch was 160 ± 6.4 μg (*n* = 5). After insertion into the skin, the CS matrix became hydrated and swelled upon contact with skin moisture, which facilitated nanovaccine diffusion from the MNs and led to the initial release of approximately 6% of the nanovaccine content (Figure 7b). Only approximately 8% of GNP–OVA was released from the CS MNs over 7 days, suggesting that the majority of the antigen was retained within the MN matrix.

**Figure 7:**
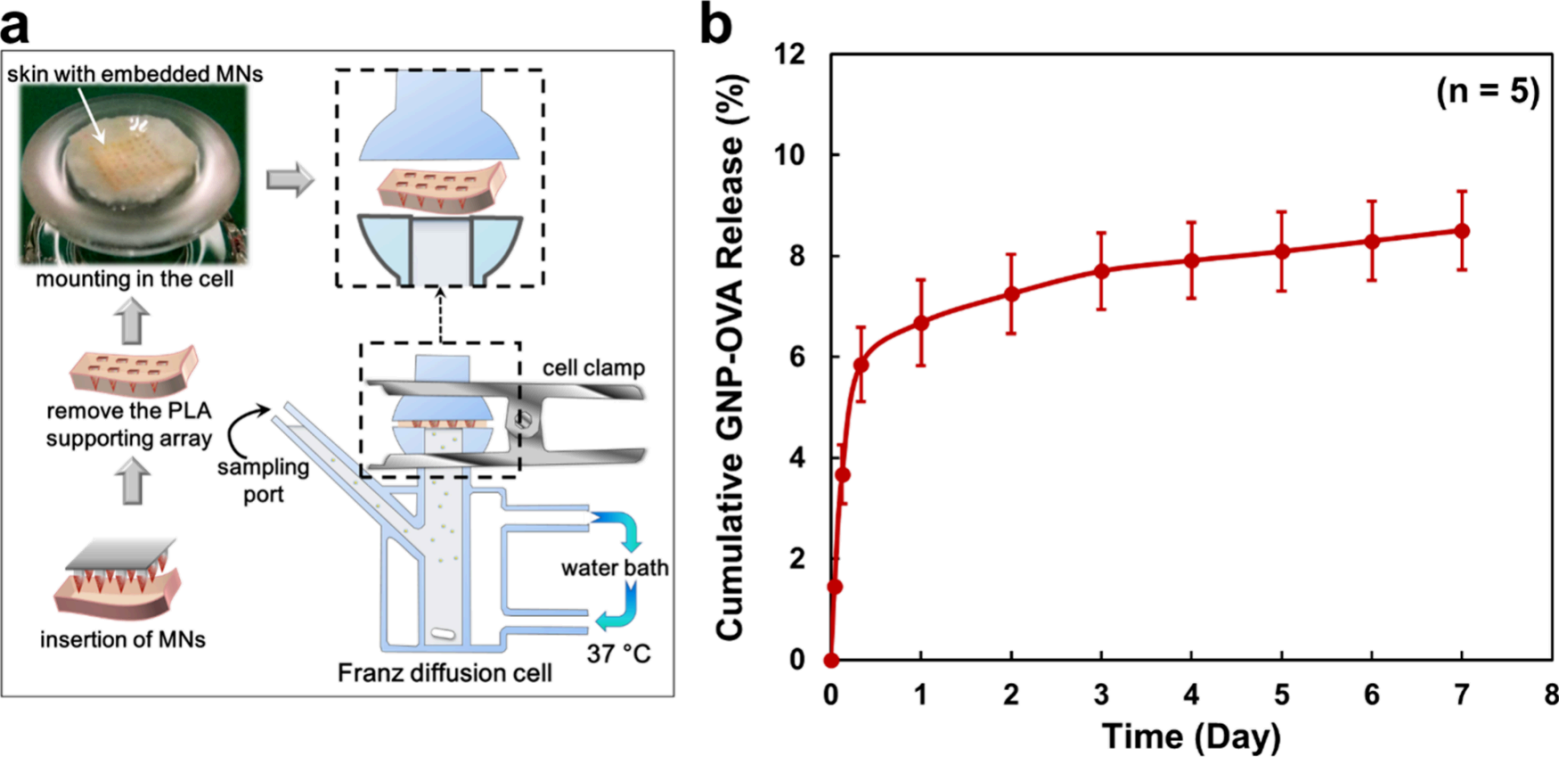
In vitro release of GNP–OVA from CS MNs (explored using a Franz diffusion cell system). (a) Schematics of MN insertion and the Franz diffusion cell system. (b) Cumulative release profile of GNP–OVA over 7 days. Data are presented as mean ± standard deviation (*n* = 5).

Overall, these results indicate that CS MNs can serve as an intradermal depot, enabling prolonged antigen persistence and thereby supporting extended immune stimulation. Notably, the aforementioned experiment involved diffusion-controlled antigen release under enzyme-free conditions. However, in vivo antigen release from CS MNs may be accelerated by the enzymatic degradation of CS (primarily mediated by lysozyme) in combination with macrophage-driven biomaterial degradation and interstitial fluid exchange, which collectively contribute to a faster release profile.

### 3.7 GNP–OVA Retention in Rat Skin

To determine whether CS MNs can prolong antigen retention within the skin, IVIS imaging was used to dynamically monitor the fluorescence signal of GNP–OVA–Cy7 at the application site and to compare the fluorescence signal intensities between subcutaneous injection and MN treatment (Figure 8).

**Figure 8:**
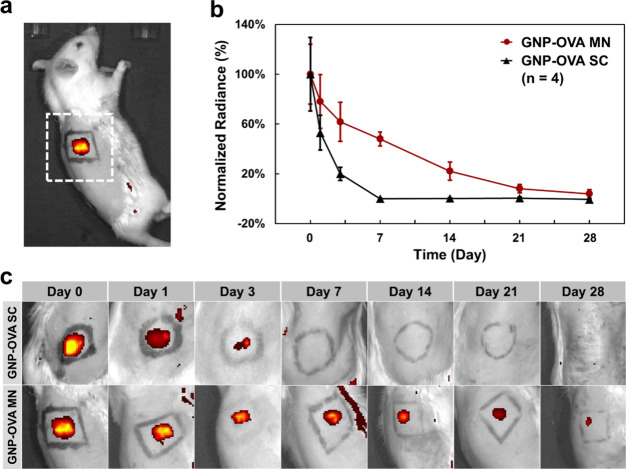
In vivo tracking of GNP–OVA retention in rat skin. (a) Representative IVIS fluorescence image depicting the ROI at the dorsal skin. (b) Quantitative analysis of fluorescence intensity at the administration site over 28 days for GNP–OVA delivered through MN and subcutaneous injection. (c) Representative IVIS images of the administration sites at different times (Days 0–28) for GNP–OVA delivered through subcutaneous injection and MNs. Data are presented as mean ± standard deviation (*n* = 4).

The results indicated that the fluorescent signal of GNP–OVA MNs gradually weakened but remained detectable for up to 28 days, whereas the signal from the subcutaneously injected GNP–OVA rapidly diminished to an undetectable level after 3 days (Figure 8b,c). These results were attributable to the depot effect of CS MNs, which function as antigen reservoirs that gradually release their payload. Compared with conventional subcutaneous injection, MN-mediated delivery is associated with markedly longer antigen persistence in the skin, ensuring sustained exposure to abundant APCs. This effect may facilitate antigen uptake and processing, ultimately leading to enhanced adaptive immune activation.

### 3.8 Immune Cell Recruitment at the MN Insertion Site

To determine whether CS-MN-mediated delivery establishes an immunostimulatory microenvironment, we examined skin histological sections at the MN insertion site (Figure 9) and compared immune cell recruitment among GNP–OVA MNs, CS MNs without OVA, and a sham control, in which nonimplantable PLA MNs were inserted and immediately removed without leaving any material in the skin. Upon disruption of the skin barrier by the MNs, the innate immune system was activated, leading to the recruitment of innate immune cells to the insertion site.^46,47^ In contrast to the sham control, the CS MNs without OVA and GNP–OVA MNs demonstrated clear embedding into the skin (indicated by the black dashed lines); however, none of the MNs exhibited discernible immune cell recruitment on Day 0 (approximately 1 h after insertion; Figure 9a).

**Figure 9:**
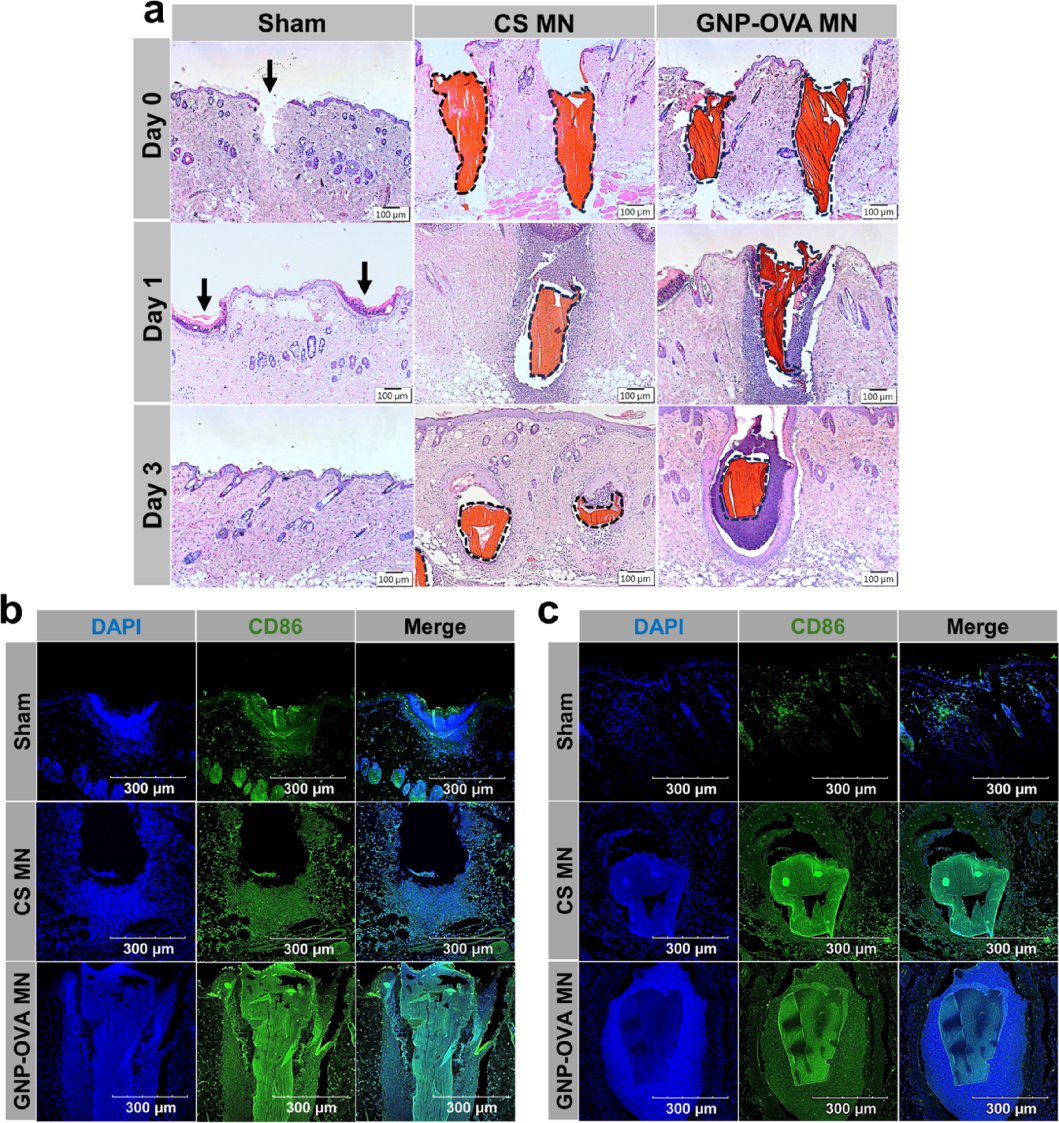
Histological and IF analyses of skin tissues after the insertion of PLA MNs (sham control), CS MNs without OVA, and GNP–OVA MNs. (a) Representative images of H&E-stained skin sections on Day 0 (approximately 1 h), Day 1, and Day 3 after MN insertion. Embedded MNs are outlined by black dashed lines, and the MN insertion sites are indicated by arrows. (b, c) IF staining images of (b) CD86 (green) and (c) nuclei (blue, identified using DAPI) on (b) Day 1 and (c) Day 3. CD86^+^ signals indicate activated APCs. Scale bars: 100 μm for H&E staining and 300 μm for IF staining.

At 24 h after insertion (Day 1), a small number of inflammatory cells accumulated at the insertion sites of the sham control (indicated by arrows); however, these cells largely resolved by Day 3, indicating that the innate immune cells recruited by the MN insertion process only accumulated transiently (Figure 9a). Markedly greater cellular infiltration was observed for CS MNs without OVA and GNP–OVA MNs compared with the sham control. Notably, for GNP–OVA MNs, densely packed cells (blue-purple) surrounded the MN insertion sites, representing substantially stronger infiltration than that observed for the CS MNs (Figure 9a). This pronounced response was likely attributable to immune cell recruitment and activation mediated by the intrinsic adjuvant effect of CS MNs, which was further potentiated by the high immunogenicity of the delivered GNP–OVA nanovaccine.

To identify the types of infiltrating cells, adjacent tissue sections were analyzed for the expression of CD86 through IF staining. As displayed in Figure 9b,c, CD86 signals (green) were predominantly observed in DAPI-stained infiltrated cells (blue), indicating that the majority of recruited cells were activated APCs. The IF results also demonstrated that the GNP–OVA MNs consistently exhibited markedly greater infiltration of CD86^+^ immune cells compared with the CS MNs, with this response persisting for at least 7 days (Figure S6). Given that activated APCs in the skin can subsequently traffic vaccines to draining lymph nodes for antigen presentation, these histological findings suggest that GNP–OVA MNs can elicit a more robust immune response than can CS MNs without OVA.

### 3.9 Immunological Evaluation of GNP–OVA MN in SD rats

To evaluate the immunogenicity of the developed vaccine formulation, four groups of SD rats were immunized in a prime–boost schedule (Weeks 0 and 2): a group given PBS through subcutaneous injection, a group given OVA through subcutaneous injection, a group given GNP–OVA through subcutaneous injection, and a group given GNP–OVA through CS MNs. The vaccine dosage for each group was 80 μg OVA per rat. After immunization, serum samples were collected from the rats and measured for OVA-specific immunoglobulin G (IgG), IgG1, and IgG2a levels through ELISA (Figure 10). As shown in Figure 10a, although anti-OVA IgG was detectable in the serum of OVA-immunized rats, the antibody levels remained low throughout the 16-week study period, indicating that the immunogenicity of OVA alone was weak. Subcutaneous injection of GNP–OVA induced significantly higher IgG production than did OVA alone from Week 4 to Week 16 (*p* < 0.001). Notably, GNP–OVA delivered through CS MNs elicited the strongest antibody responses, resulting in markedly higher antibody levels compared with subcutaneously injected OVA and GNP–OVA from Week 2 onward and maintaining robust responses until the end of the study period (16 weeks). These results indicated that GNP–OVA conjugation enhanced the immunogenicity of OVA, which was further boosted through CS-MN-based delivery, leading to the most potent and durable antibody responses.

**Figure 10:**
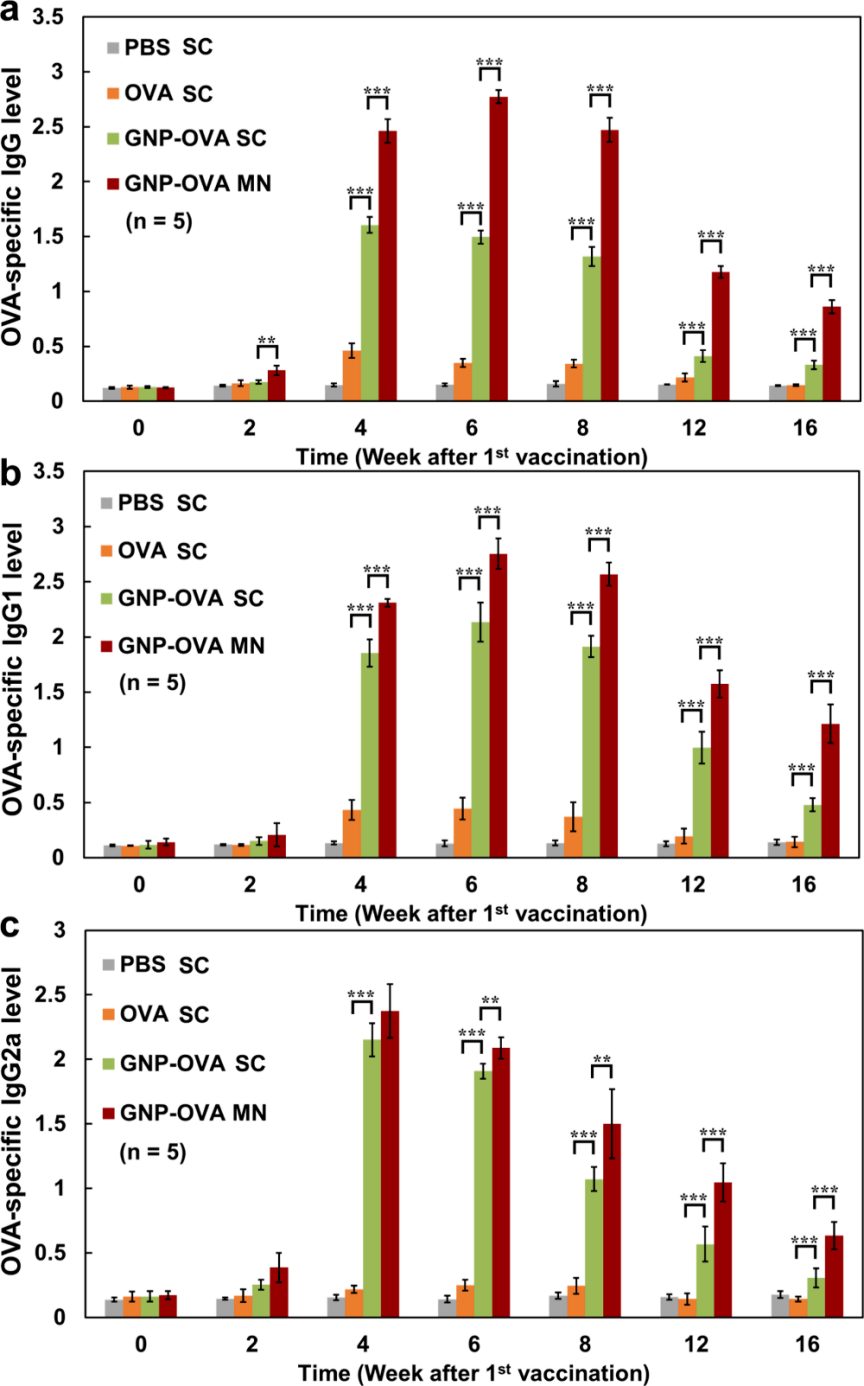
OVA-specific antibody responses induced by immunization in SD rats: (a) total IgG, (b) IgG1, and (c) IgG2a levels. SD rats were immunized through a subcutaneous injection with PBS, OVA, or GNP–OVA or through CS-MN-based delivery of GNP–OVA in accordance with a prime–boost schedule (Weeks 0 and 2). Serum samples were collected and analyzed for total IgG, IgG1, and IgG2a levels by using ELISA assays. Data are presented as mean ± standard deviation (*n* = 5). *p < 0.05; **p < 0.01; ***p < 0.001.

Overall, the superior humoral responses of GNP–OVA delivered through CS MNs can be mechanistically explained by the ability of GNP–OVA MNs to facilitate sustained antigen release at the dermal site, where APCs are abundant, thereby promoting prolonged and localized immune stimulation. In addition, conjugation of OVA onto GNPs mimics viral structural features and enables multivalent antigen display to APCs, thereby facilitating DC maturation and antigen presentation.^35^ This antigen depot-mediated persistence conferred by CS MNs, together with the multivalent interaction between APCs and GNP–OVA, may provide a mechanistic basis for the strength and durability of the antibody responses elicited by GNP–OVA MNs.

Subclass analysis of OVA-specific antibodies revealed slightly different kinetics between IgG1 and IgG2a responses induced by GNP–OVA MNs. The IgG1 levels (Figure 10b) closely aligned with the total IgG levels (Figure 10a), demonstrating a rapid increase after the booster dose was administered, reaching peak values at Week 6 before marginally declining. Nevertheless, the IgG1 levels induced by GNP–OVA MNs remained substantially higher than those induced by the other treatments throughout the study period. By contrast, the IgG2a levels induced by GNP–OVA MNs (Figure 10c) reached their peak values in Week 4 and then gradually declined. Notably, CS-MN-mediated delivery of GNP–OVA consistently enhanced both IgG1 and IgG2a responses, eliciting the strongest antibody production across all subclasses.

IgG1 is generally associated with T helper 2- (Th2-) biased responses, whereas IgG2a is indicative of T helper 1- (Th1-) associated immunity. In this study, the IgG1 and IgG2 levels induced by GNP–OVA MNs peaked at different weeks (Weeks 6 and 4, respectively), suggesting temporal differences in Th2- and Th1-associated pathways. The early rise of IgG2a levels induced by both the subcutaneously injected GNP–OVA and CS-MN-delivered GNP–OVA likely reflects the particulate and virus-mimicking nature of GNP–OVA, which facilitates APC engagement and promotes Th1-associated immunity. Subsequently, the prolonged retention of GNP–OVA in draining lymph nodes, combined with its sustained release from the CS-based MN matrix, may support extended antigen presentation, thereby amplifying Th2-associated humoral responses. These findings indicate that both Th1- and Th2-associated pathways are enhanced and prolonged, collectively contributing to the potent and durable antibody responses elicited by GNP–OVA MNs.

The robust enhancement of both IgG1 and IgG2a levels through the CS-MN-based delivery of the GNP–OVA nanovaccine indicates that the proposed dual strategy did not strongly bias T helper cell polarization but rather promoted a balanced and prolonged immune response, which can be attributed to the virus-mimicking and self-adjuvanting properties of GNPs and the intradermal depot effect provided by CS MNs. Such balanced activation of Th1 and Th2 is desirable in vaccine development because it facilitates both humoral and cellular immune protection.

After MN insertion, mild and transient erythema was observed at the administration site (Day 0, Figure S7), consistent with the expected temporary disruption of the stratum corneum. These local reactions gradually resolved within 24–48 h, and the skin surface appeared almost completely healed by Day 3, showing no signs of infection, ulceration, or scarring (Figure S7). No abnormal behavior or other adverse clinical signs were observed in any animal throughout the study period.

No significant body weight loss was detected for the groups receiving GNP–OVA through subcutaneous injection or CS MNs (Figure S8). Moreover, the body weight profiles of these groups were comparable to that of the PBS control group (*p* > 0.05), suggesting the absence of apparent systemic toxicity. These findings collectively indicate that the GNP–OVA-loaded MN vaccination system is well tolerated in vivo, with strong local biocompatibility and no observable systemic adverse effects.

## 4 Conclusions

In this study, we developed an implantable CS MN system loaded with a GNP-based nanovaccine as a highly immunogenic formulation for subunit vaccines. This platform integrates the virus-mimicking and self-adjuvanting properties of GNP–antigen conjugates with the depot effect provided by MN-mediated intradermal delivery. The resulting formulation enables efficient and sustained antigen delivery within the dermis and draining lymph nodes, thereby activating both Th1- and Th2-associated immune pathways and ultimately eliciting a robust, durable, and balanced immune response. Overall, our findings elucidate the mechanisms underlying CS-MN-assisted nanovaccine immunogenicity, providing valuable insights for the rational design of next-generation subunit vaccines. Moreover, we demonstrate the immunological benefits of integrating GNP–antigen conjugation with CS-MN-based delivery, highlighting the translational potential of this approach as a minimally invasive and adjuvant-sparing strategy.

## Supplementary Material



## Data Availability

The data that support the findings of this study are available from the corresponding author upon reasonable request.

## Notes

Synthesis method of Cy5-OVA, Cy7-OVA, and Cy3-GNP-OVA-Cy5; semiquantitative confocal image analysis method for assessing codelivery; schematic illustration of GNP-OVA MN fabrication process (Figure S1); size distribution of GNP, GNP-PEG and GNP-OVA (Figure S2); cell viability assessment (Figure S3); quantitative percentages of CD86 and MHC-II positive cells (Figure S4); semiquantitative analysis of the spatial correlation between OVA and GNP in DCs (Figure S5); immunofluorescence staining images of CD86 and nuclei in skin tissues collected at Day 7 (Figure S6); representative photographs of the skin at the MN application site on Day 0, Day 1, and Day 3 (Figure S7); SD rat weight monitoring during immunization (Figure S8); reaction concentrations of EDC and NHS for the synthesis of GNP-OVA conjugates (Table S1); quantification of the amount of OVA conjugated on GNPs (Table S2) (PDF)
