# Supplementary material for: Augmenting Subunit-Vaccine-Induced Immunity through a Dual Strategy of Gold Nanoparticle Conjugation and Chitosan Microneedle-Mediated Sustained Delivery
Source: ACS Appl Mater Interfaces. 2025 Dec 19;18(1):553–65. doi: 10.1021/acsami.5c20082 (PMC12781117; doi:10.1021/acsami.5c20082)
Supplement: Supplementary file 1 [file am5c20082_si_001.pdf]

Supporting Information for

Augmenting Subunit Vaccine-Induced Immunity through a Dual Strategy of Gold Nanoparticle Conjugation and Chitosan Microneedle-Mediated Sustained Delivery

Zih-Yao Lin[1], Yi-Lun Chen[1], Cheng-Lin Wu[2,3], Yu-Hung Chen[4], Mei-Chin Chen[1]*

[1]Department of Chemical Engineering, National Cheng Kung University, Tainan, Taiwan.
[2]Department of Pathology, National Cheng Kung University Hospital, College of Medicine, National Cheng Kung University, Tainan, Taiwan.
[3]Institute of Clinical Medicine, College of Medicine, National Cheng Kung University, Tainan, Taiwan.
[4]School of Medicine, College of Medicine, National Cheng Kung University, Tainan, Taiwan.

***Correspondence to:**
Mei-Chin Chen, PhD
Professor
Department of Chemical Engineering
National Cheng Kung University
Tainan, Taiwan 70101
Tel: +886-6-275-7575 # 62696
Fax: +886-6-234-4496
E-mail: kokola@mail.ncku.edu.tw

Methods

Synthesis of Cy5-OVA, Cy7-OVA, and Cy3-GNP-OVA-Cy5

The fluorescent labels used in **Section 2.6** and **Section 2.7**, including sulfo-Cy3 amine, Cy5-SE triethylamine salt and Cy7-NHS ester were purchased from MedChemExpress (Monmouth Junction, NJ, USA).

Cy5-SE triethylamine salt and Cy7-NHS ester were conjugated to OVA following the manufacturers' instructions. Briefly, 4 mg of OVA was dissolved in 1 mL of PBS (pH 8.0), and 1 mg of Cy5-SE triethylamine salt or Cy7-NHS ester was dissolved in 100 µL of DMSO. Subsequently, 38 µL of the Cy5-SE solution or 30 µL of the Cy7-NHS solution was added to 1 mL of the OVA solution and allowed to react for 1 h in the dark under gentle shaking. The resulting Cy5-OVA or Cy7-OVA conjugates were dialyzed against deionized water using a dialysis membrane (MWCO 3 kDa) for three days to remove unreacted fluorescent dyes.

To synthesize Cy3-GNP-OVA-Cy5 conjugates, sulfo-Cy3 amine was conjugated to GNP-PEG via the EDC/NHS coupling reaction described in **Section 2.3** with slight modifications. After the activation step, 20 µL of sulfo-Cy3 amine solution (0.3 mg/mL) was added to the GNP-PEG solution and allowed to react for 3 min. Subsequently, 200 µL of Cy5-OVA solution (0.64 mg/mL) was added to the reaction mixture and incubated for 4 h to obtain the Cy3-GNP-OVA-Cy5 conjugates.

Co-delivery semi-quantitative analysis of confocal images

We employed Pearson's correlation coefficient (PCC) to quantify the pixel-level linear correlation between the OVA and GNP fluorescence channels, while minimizing the influence of background variability.[S1] PCC is a widely accepted metric for assessing the degree to which intensity variations in one channel coincide spatially with those in another.[S2] PCC analyses were performed in ImageJ using the JACoP plugin. For each group, 10–20 cells per imaging field were evaluated across four independent fields under identical thresholding parameters. Mean PCC values obtained from these fields were presented as mean ± standard deviation.

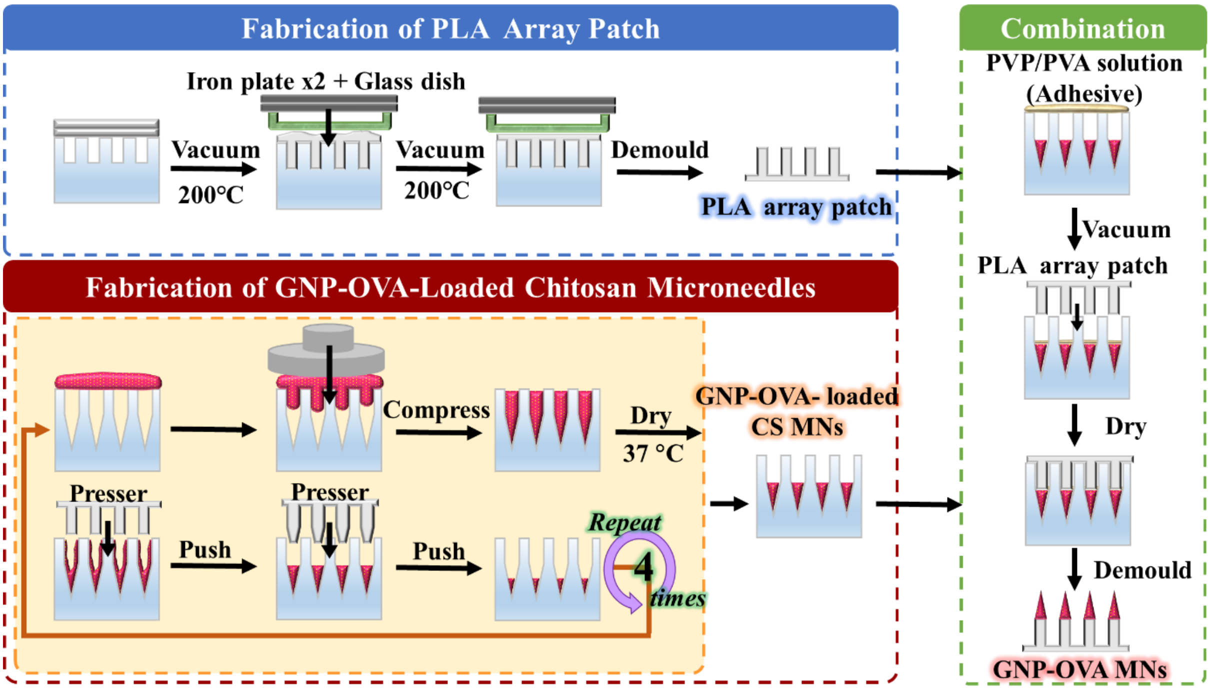

Figure S1. Illustration of GNP-OVA MN fabrication process. PLA filaments were melted and injected to the supporting array mold with vacuum casting process to form PLA array patch. The GNP-OVA loaded chitosan MNs were fabricated using compression molding and attached to PLA array patch by adhesive integration.

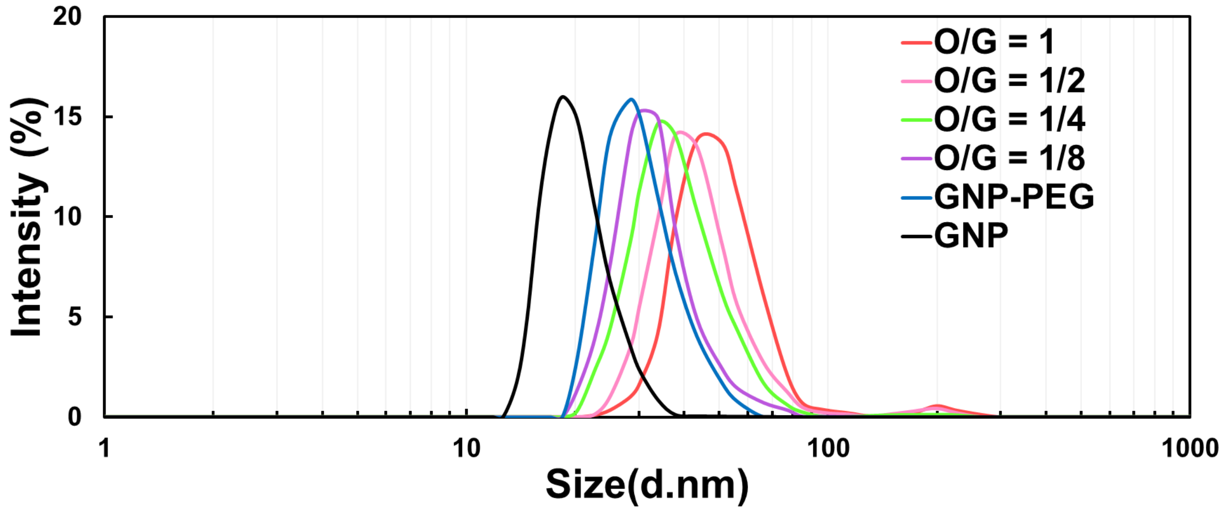

Figure S2. Size distribution of GNP, GNP-PEG, and GNP-OVA with different O/G ratios.

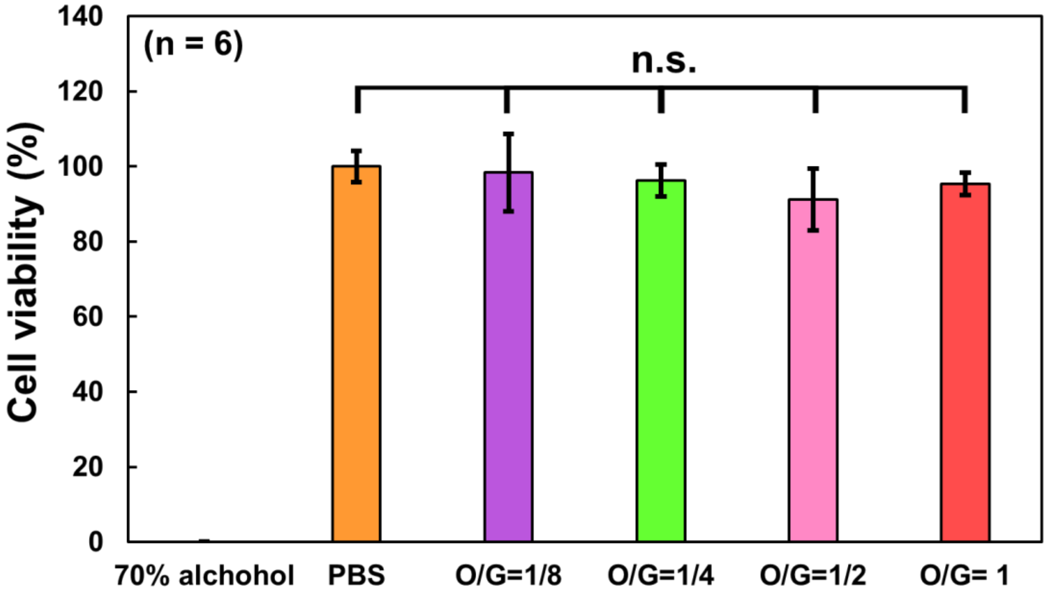

Figure S3. Cell viability assessment of GNP-OVA with different O/G ratios (n = 6). PrestoBlue assay was utilized to examine the cytocompatibility of GNP-OVA with a fixed GNP dose (400 µg/mL) and incubation period (12 h).

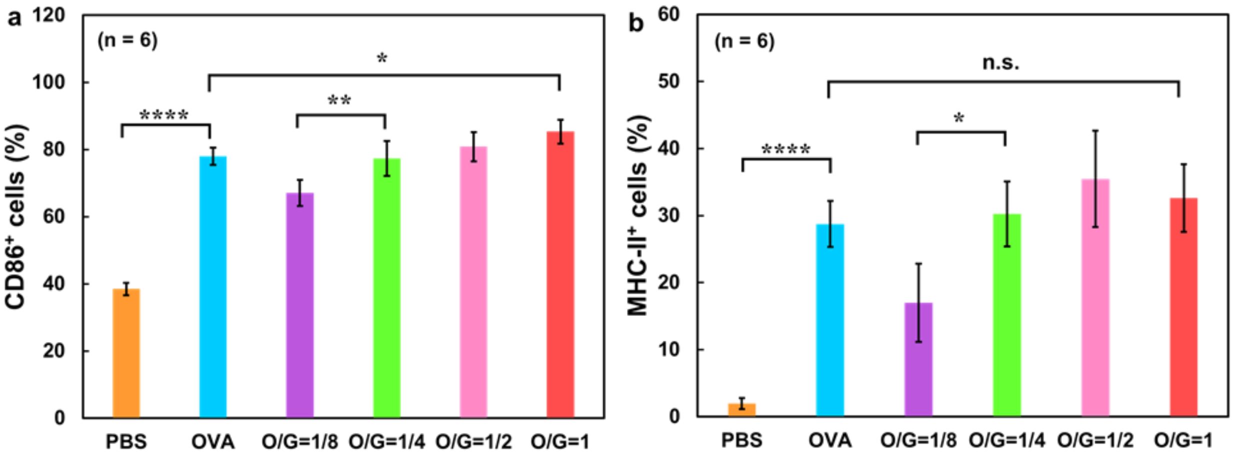

Figure S4. Flow cytometric analysis of (a) CD86 and (b) MHC-II positive cell percentages in DC2.4 cells treated with free OVA and GNP-OVA nanovaccines carrying different O/G ratios. Data are shown as mean ± SD (n = 6). Statistical significance: $p < 0.05$ (*), $p < 0.01$ (**), $p < 0.0001$ (****).

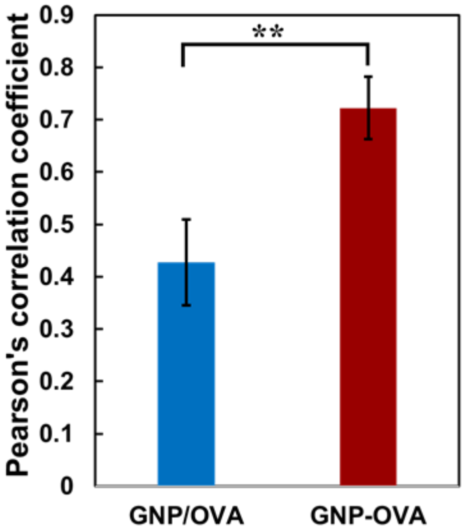

Figure S5. Semi-quantitative analysis of the spatial correlation between OVA and GNP. Pearson's correlation coefficient (PCC) was calculated to assess the pixel-based linear correlation between the OVA and GNP fluorescence channels in DC2.4 cells (n = 4 fields per group). Data are presented as mean ± SD. Statistical significance was determined at $p < 0.01$ (**).

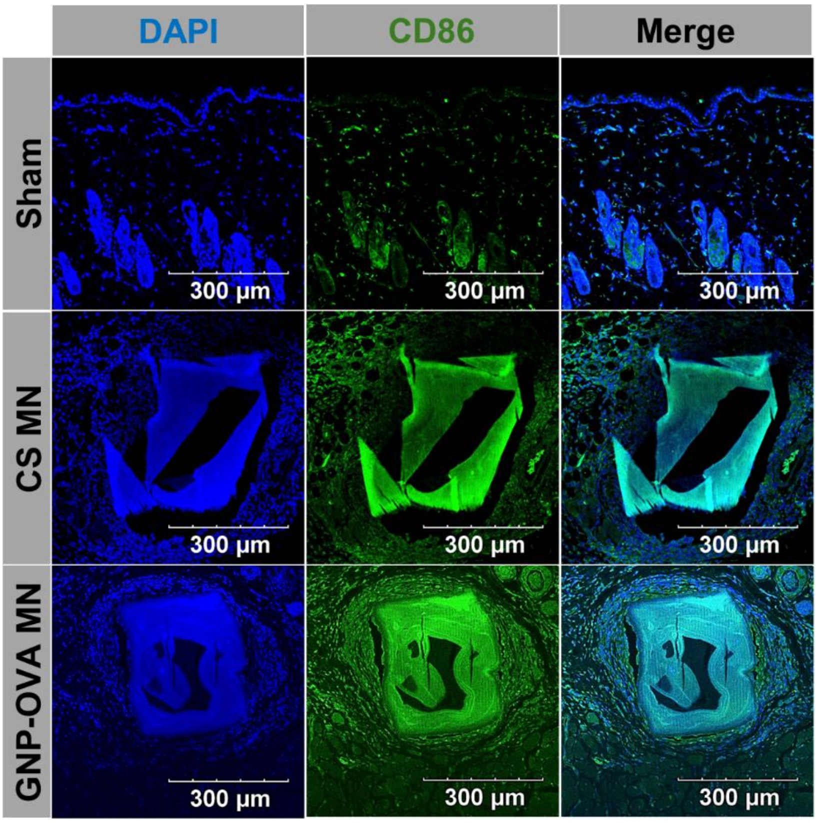

Figure S6. Immunofluorescence analysis of CD86 (green) and nuclei (DAPI, blue) in skin tissues collected at Day 7 after insertion of PLA MNs (Sham), CS MNs, or GNP-OVA MNs. Scale bars: 300 μm.

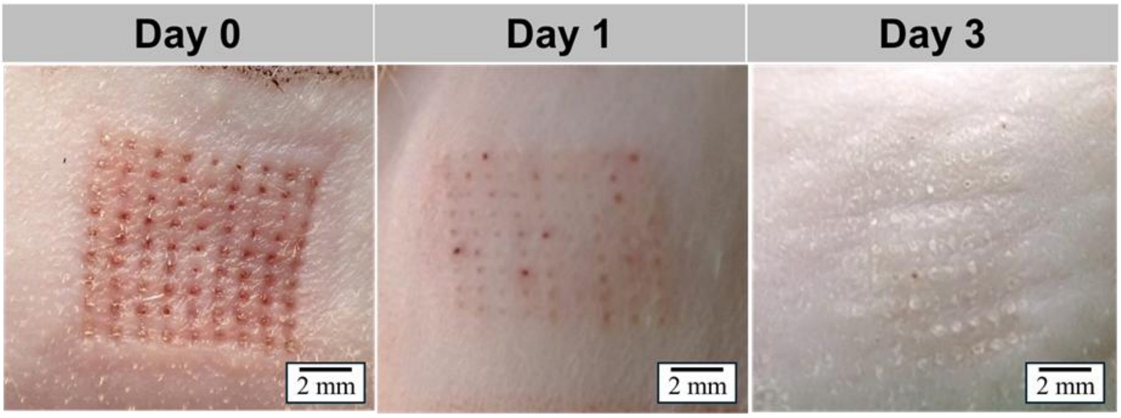

Figure S7. Representative photographs of the skin at the microneedle application site on Day 0, Day 1, and Day 3. Local erythema diminished progressively, and the skin recovered almost completely by Day 3 without infection, ulceration, or scarring. Scale bar: 2 mm.

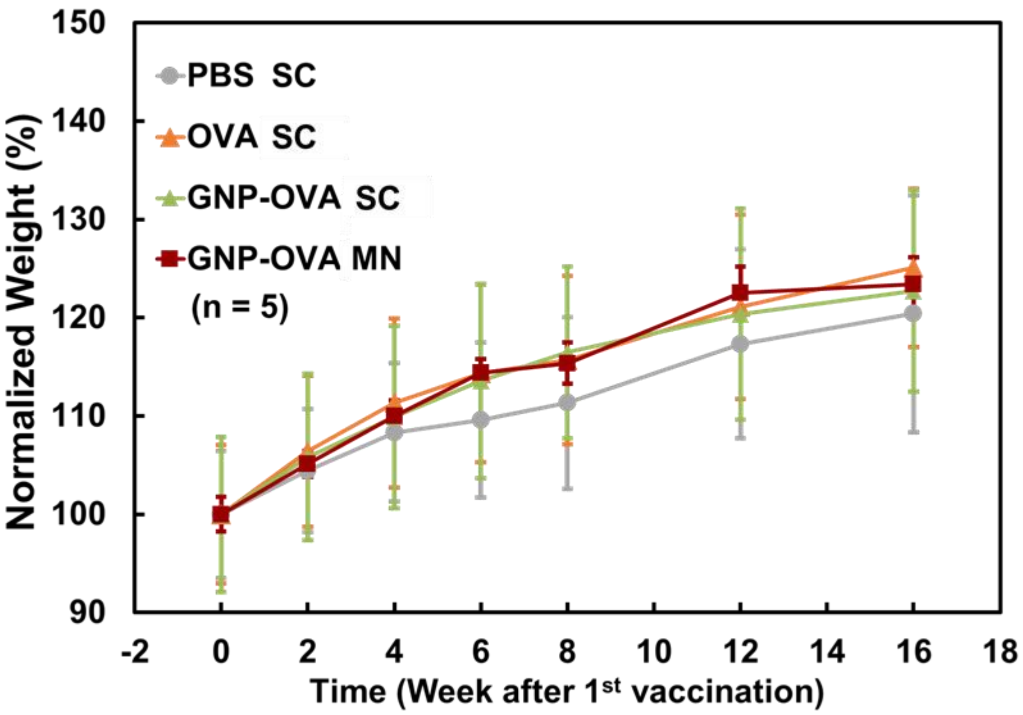

Figure S8. SD rat weight monitoring during immunization study. SD rats were immunized by subcutaneous (SC) injection with PBS, OVA, or GNP-OVA, or by application of GNP-OVA-loaded MNs (GNP-OVA MN), following a prime-boost schedule (Week 0 and Week 2).

Table S1. Reaction concentrations of EDC and NHS solutions used to graft different amounts of OVA onto GNPs for the synthesis of GNP-OVA conjugates. O/G: OVA/GNP conjugation ratio

	**EDC**	**NHS**	**OVA**
**O/G= 1**	1.5×10^{-3} M	3×10^{-3} M	128 μg/ml
**O/G= 1/2**	6×10^{-4} M	1.2×10^{-3} M	64 μg/ml
**O/G= 1/4**	3×10^{-4} M	6×10^{-4} M	32 μg/ml
**O/G= 1/8**	1.5×10^{-4} M	3×10^{-4} M	16 μg/ml

Table S2. Quantification of grafted OVA amounts on GNPs at different OVA feeding concentrations (OVA_{in}, n = 8). The conjugated OVA amount (OVA_{conj}) was calculated by subtracting the unreacted OVA in the supernatant (OVA_{sup}), determined by bicinchoninic acid (BCA) assay, from the initial OVA feed (OVA_{in}). O/G: OVA/GNP conjugation ratio

	$\mathbf{OVA_{in}}$ (μg/ml)	$\mathbf{OVA_{sup}}$ (μg/ml)	$\mathbf{OVA_{conj}}$ (μg/ml)	**GNP** (μg/ml)
**O/G= 1**	128	43.3 ± 5.7	84.7 ± 5.7	88
**O/G= 1/2**	64	19.3 ± 3.4	44.7 ± 3.4	88
**O/G= 1/4**	32	10.1 ± 0.8	21.9 ± 0.8	88
**O/G= 1/8**	16	5.4 ± 0.9	10.6 ± 0.9	88

References

[S1] Liang, X. H.; Nichols, J. G.; De Hoyos, C. L.; Sun, H.; Zhang, L.; Crooke, S. T. Golgi-58K Can Re-Localize to Late Endosomes upon Cellular Uptake of PS-ASOs and Facilitates Endosomal Release of ASOs. *Nucleic Acids Research* **2021**, *49* (14), 8277–8293.

[S2] Nam, G.; Yeon, H. R.; Park, H. B.; Chang, H.; Kim, J. H.; Cho, B. K.; Jung, H.; Yi, E. C.; Kim, S.; An, J. Y.; Lee, J. E.; Lee, Y.; Lee, S.; Lim, H.; Shon, W. J.; Hwang, E. M.; Ryu, H.; Chang, J.; Choi, K.; Choi, E. Y. CD99-Mediated Immunological Synapse Formation Potentiates CAR-T Cell Function. *Nat Commun* **2025**, *16* (1), 7987.
